# Supplementary material for: Myocardial TRPC6-mediated Zn2+ influx induces beneficial positive inotropy through β-adrenoceptors
Source: Nat Commun. 2022 Oct 26;13:6374. doi: 10.1038/s41467-022-34194-9 (PMC9606288; doi:10.1038/s41467-022-34194-9)
Supplement: Supplementary file 1 — Supplementary Information [file 41467_2022_34194_MOESM1_ESM.doc]

**Supplementary Information for**

**Myocardial TRPC6-mediated Zn^2+^ influx induces beneficial positive inotropy through β-adrenoceptors**

**Supplementary Figures**


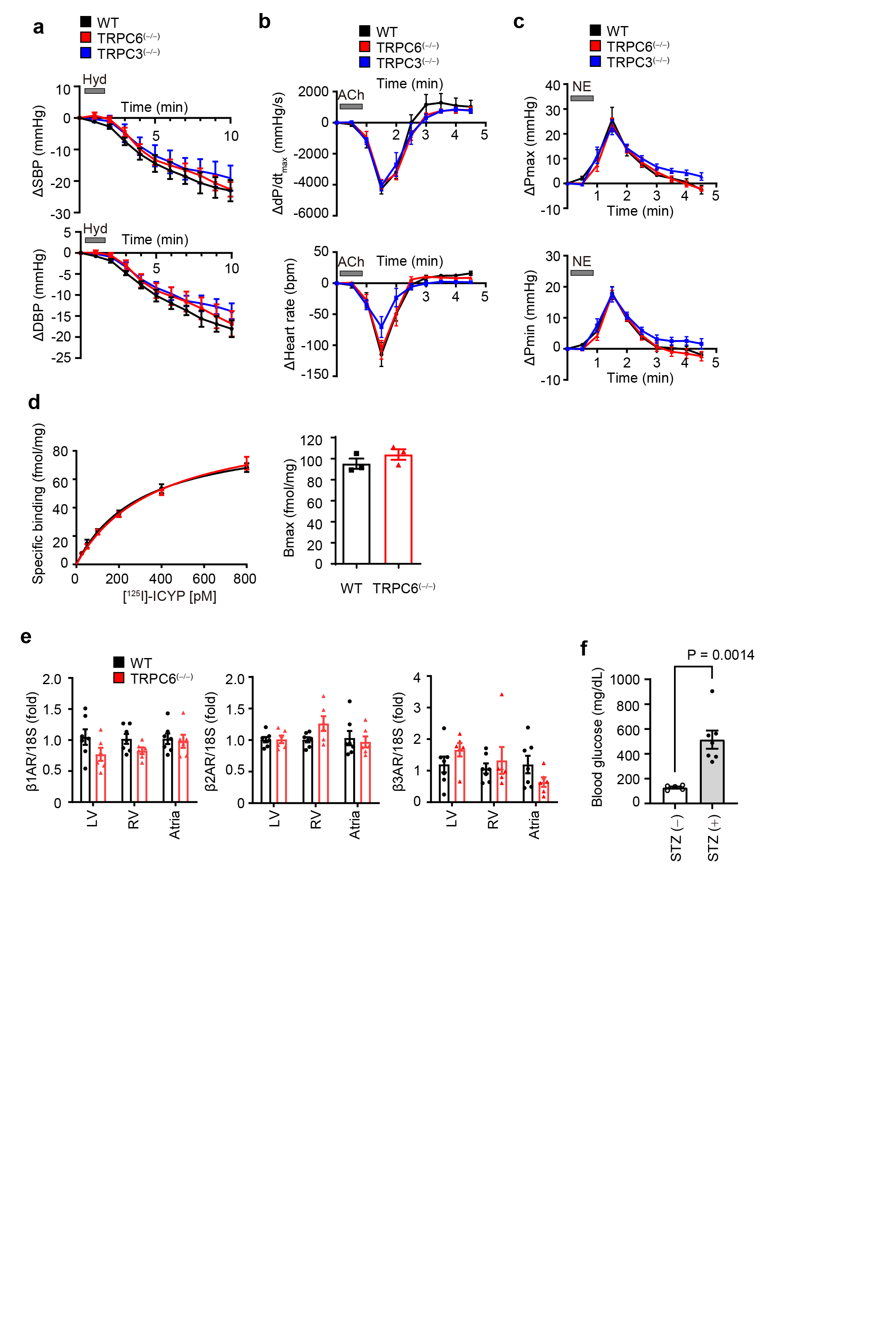


**Supplementary Figure 1.** **Expression of βAR is not altered in TRPC6-deficient mouse hearts.**

(**a**) Time courses of decreases in systolic aortic pressure (ΔPmax; left) and diastolic aortic pressure (ΔPmin; right) induced by hydralazine in mice (129/sv). WT, n=5; TRPC6^(-/-)^, n=5; TRPC3^(-/-)^, n=5. (**b**) Time courses of ΔdP/dt max (top) and Δheart rate (bottom) following *i.v.* administration of acetylcholine (ACh; 0.5 mg/kg, 1.8 mL/h) in mice (WT, n=6; TRPC6^(-/-)^, n=6; TRPC3^(-/-)^, n=5). (**c**) Time courses of increases in systolic aortic pressure (ΔPmax; top) and diastolic aortic pressure (ΔPmin; bottom) following *i.v.* administration of norepinephrine (NE; 0.05 mg/kg, 1.8 mL/h) in mice (129/sv). WT, n=6; TRPC6^(-/-)^, n=6; TRPC3^(-/-)^, n=6. (**d**) Expression levels and binding affinities of βARs in WT and TRPC6^(-/-)^ hearts. WT, n=3; TRPC6^(-/-)^, n=3. (**e**) mRNA expression levels of βAR subtypes in LV, RV and atria. mRNA levels were measured by RT-PCR. WT, n=7; TRPC6^(-/-)^, n=6. (**f**) Blood glucose levels at 4 weeks after streptozotocin (STZ) administration. Mice (129/Sv) were injected with STZ (50 mg/kg) intraperitoneally for 5 successive days. STZ(-), n=5; STZ(+), n=7. Data are shown as mean±SEM. **P<0.01 by the unpaired t-test (two-sided).


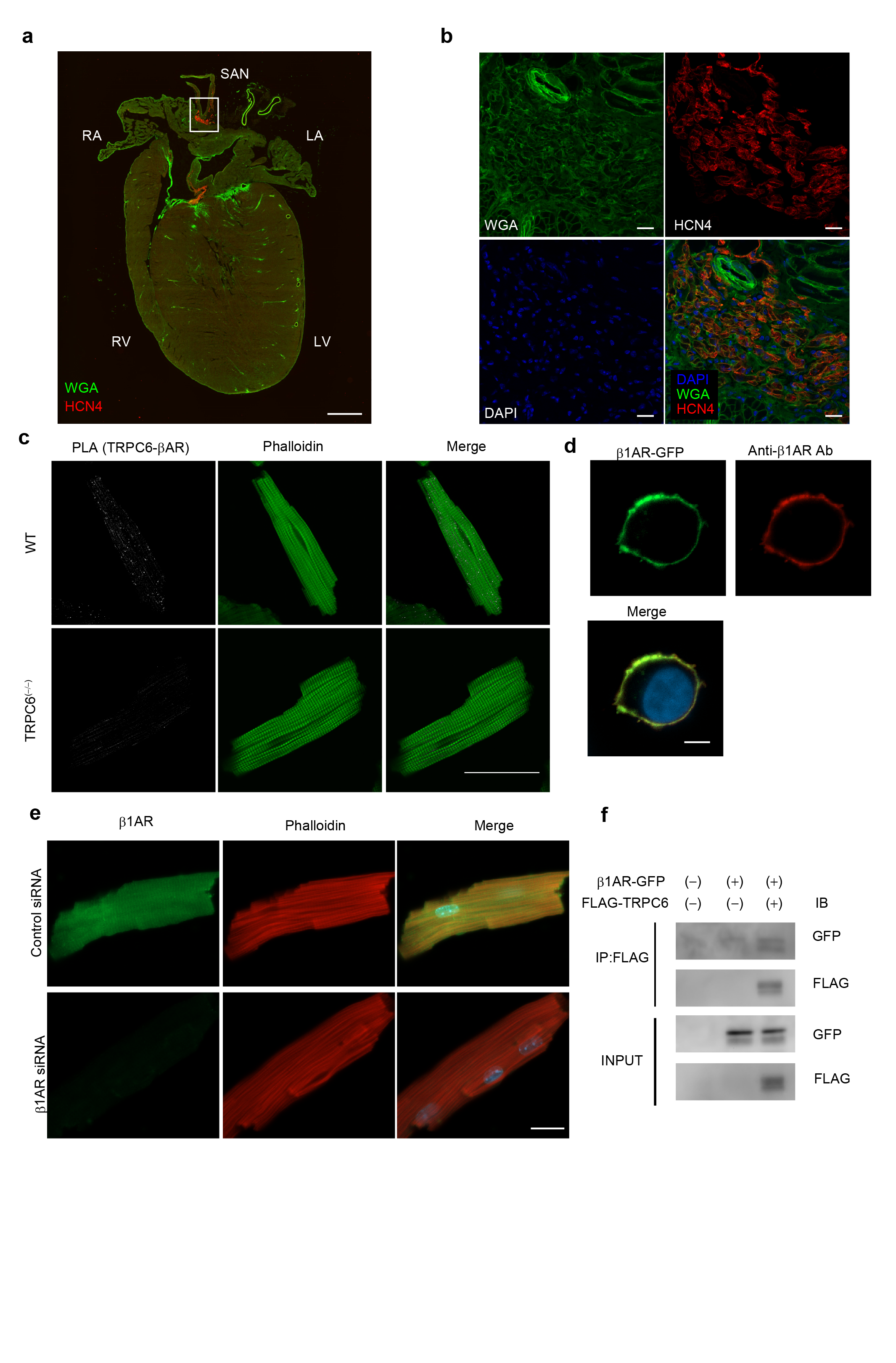


**Supplementary Figure 2. TRPC6 co-localizes and physically interacts with β_1_AR.**

(**a**) Fluorescence image of a whole heart section stained with Alexa Fluor 488–conjugated wheat germ agglutinin (WGA; green) and hyperpolarization-activated cyclic nucleotide-gated channel 4 (HCN4; red). Boxed area is magnified in (**b**). Scale bar, 1 mm. SAN, sinoatrial node; RA, right atrium; RV, right ventricle; LA, left atrium; LV, left ventricle. (**b**) Magnified images of the SAN region in (**a**). Green, WGA; red, HCN4; blue, DAPI. Scale bars, 20 μm. (**c**) Representative PLA images of TRPC6 and β_1_AR in adult cardiomyocytes isolated from WT or TRPC6^(-/-)^ mice (129/sv). PLA signals are visualized by white spots. Cells were counterstained with phalloidin (green). Scale bar, 20 μm. (**d**) Fluorescence and immunostaining images of HEK293 cells expressing β_1_AR-GFP. Scale bars, 20 μm (**e**) Fluorescence image of β_1_AR-silenced cardiomyocytes stained with β_1_AR (green) and phalloidin (red). Scale bar, 20 μm. (**f**) Immunoprecipitation assay using HEK293 cells expressing FLAG-TRPC6 and β_1_AR-EGFP. Images are representative of three independent experiments (a-f).

.


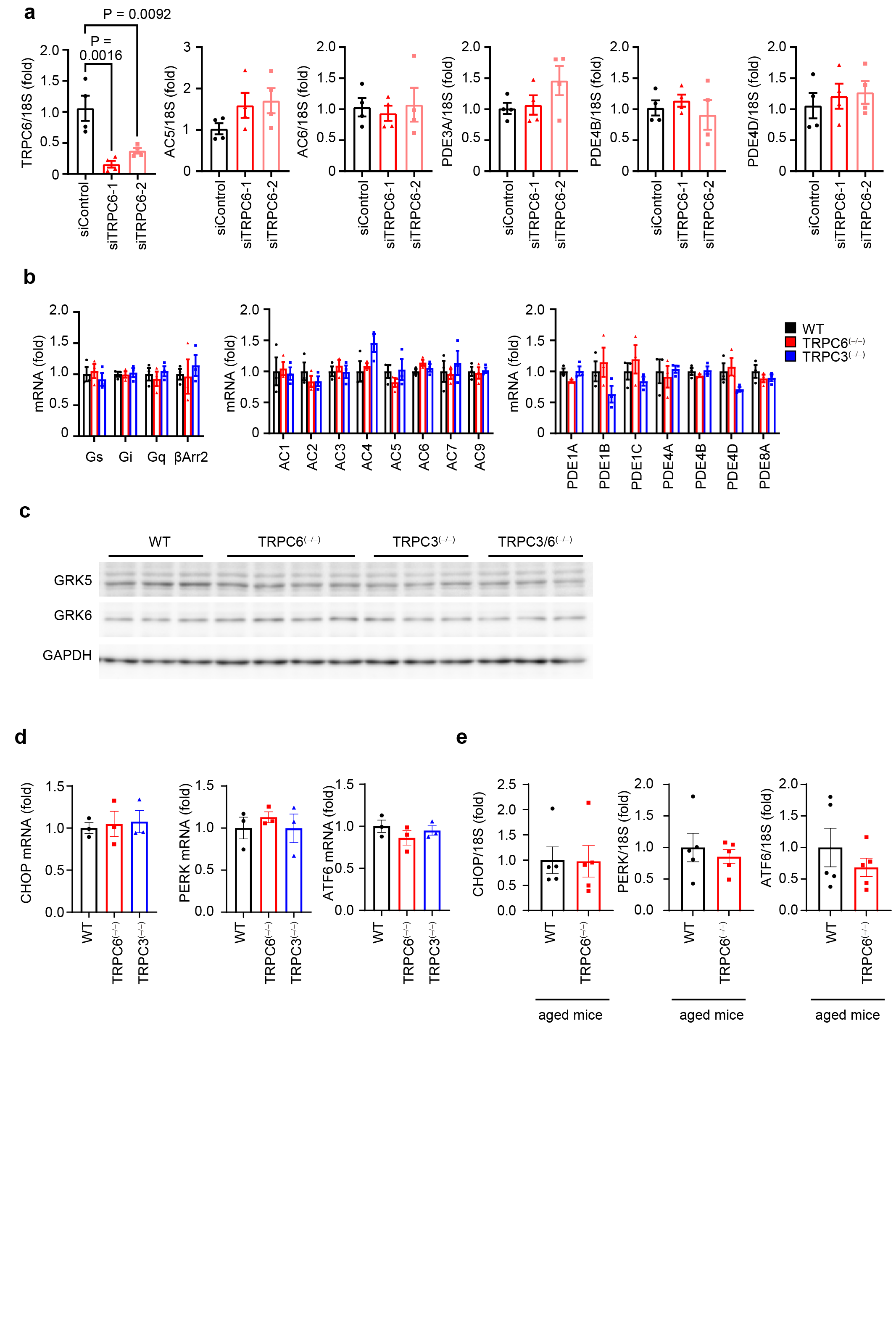


**Supplementary Figure 3. TRPC6 deletion does not affect the expression levels of cAMP-related factors and endoplasmic reticulum stress markers in mouse hearts and neonatal rat cardiomyocytes (NRCMs).**

(**a**) mRNA expression levels of TRPC6, adenylate cyclase (AC) and phosphodiesterase (PDE) isoforms in siRNA-treated NRCMs. n=4 each group. (**b**) mRNA expression levels of heterotrimeric G proteins, β-arrestin 2 (βArr2) and AC and PDE isoforms in adult mouse (129/sv) hearts. n=3 each group. (**c**) Protein expression levels of G protein–coupled receptor kinase (GRK) subtypes in mouse (129/sv) hearts. WT, n=3; TRPC6^(-/-)^, n=4; TRPC3^(-/-)^, n=3; TRPC3/6^(-/-)^, n=3. (**d**) mRNA expression levels of CCAAT-enhancer-binding protein homologous protein (CHOP), protein kinase R-like ER kinase (PERK) and activating transcription factor 6 (ATF6) in adult mouse (129/sv) hearts analyzed by microarray. n=3 each. (**e**) mRNA expression levels of CHOP, PERK and ATF6 in hearts from old mice (129/sv) (90-weeks old). n=5 each. Data are shown as mean±SEM. **P<0.01 using one-way ANOVA followed by Tukey’s post-hoc test.


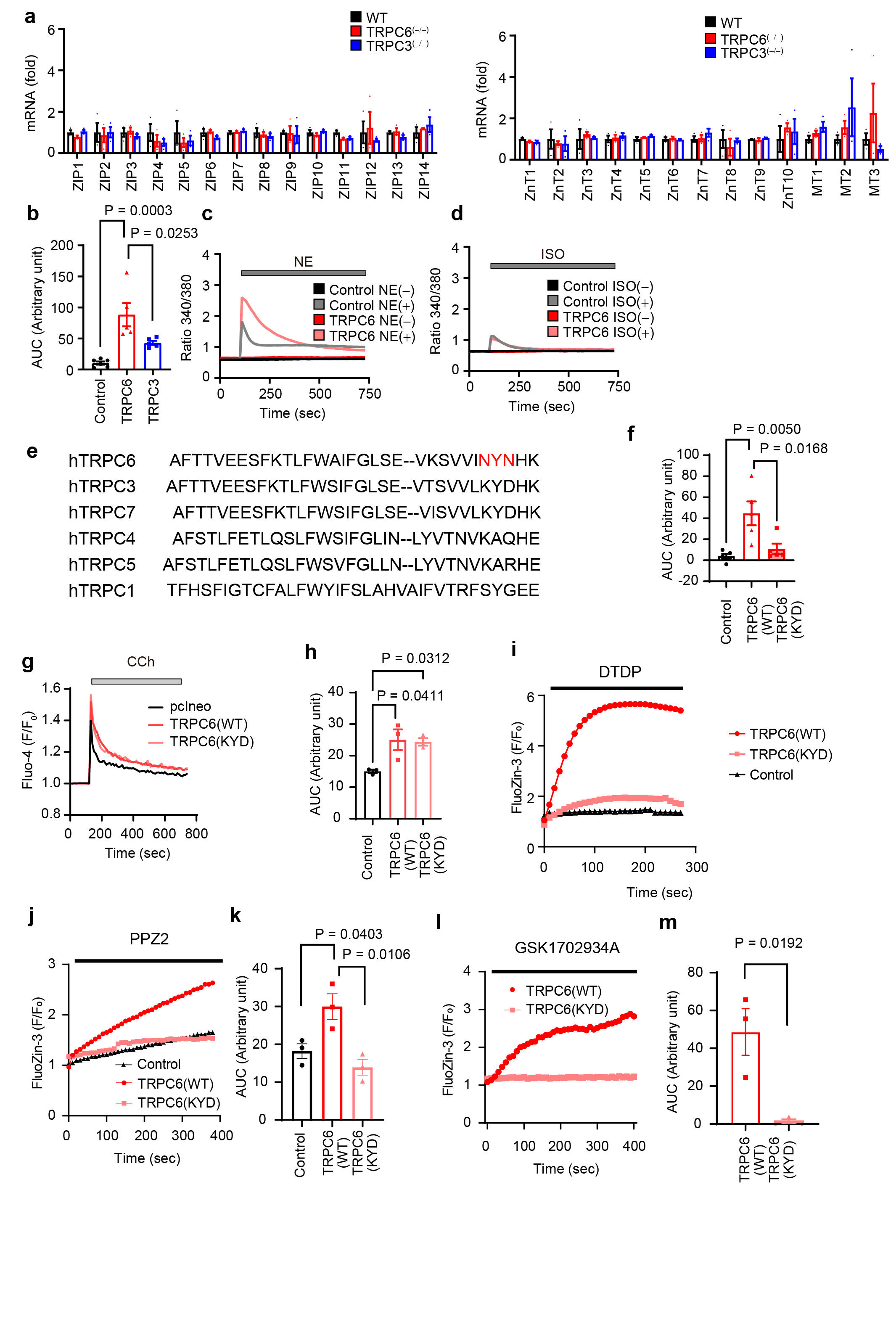


**Supplementary Figure 4. Requirement of two TRPC6-specific amino acids in Zn^2+^-selective permeability of TRPC6**

(**a**) mRNA expression levels of zinc transporters (ZIPs and ZnTs) and metallothioneins (MTs) in mouse (129/sv) hearts analyzed by microarray. n=3 each group. (**b**) Changes in intracellular Zn^2+^ concentrations in response to NE stimulation in TRPC-expressing HEK293 cells. ZnCl_2_ (50 μM) was applied to cells for 3 min before NE stimulation (10 μM). Cumulative increases in FluoZin-3 fluorescence are shown as AUC. n=5 each. (**c**) Average traces of the intracellular Ca^2+^ concentration in HEK293 cells expressing α_1A_AR and TRPC6. Cells were stimulated with NE (10 μM). n=3 each. (**d**) Average traces of the intracellular Ca^2+^ concentration in HEK293 cells expressing β_1_AR and TRPC6. Cells were stimulated with ISO (10 μM). n=4 each. (**e**) Sequence alignment of human TRPCs. Red shows TRPC6-specific sequence. (**f**) Changes in intracellular Zn^2+^ concentrations in response to NE stimulation in TRPC6 (WT or KYD)-expressing HEK293 cells. ZnCl_2_ (50 μM) was applied to cells for 3 min before NE stimulation (10 μM). Cumulative increases in FluoZin-3 fluorescence are shown as AUC. n=5 each. (**g**, **h**) Time courses of changes in intracellular Ca^2+^ concentrations in response to carbachol (CCh, 100 μM) stimulation in TRPC6 (WT or KYD)-expressing HEK293 cells (**g**). Cumulative increases in Fluo4 fluorescence are shown as AUC (**h**). n=3 each. (**i**) Increase of FluoZin-3 fluorescence (F/F_0_) in response to the application of DTDP in TRPC6 (WT or KYD)-expressing rat aortic smooth muscle cells (RAOSMCs). The horizontal bar indicates when DTDP was added. (**j**) Increase of FluoZin-3 fluorescence (F/F_0_) in response to the application of PPZ2 in TRPC6 (WT or KYD)-expressing RAOSMCs. (**k**) Cumulative increases are shown as AUC. n=3 each. (**l**) Increase of FluoZin-3 fluorescence (F/F_0_) induced by GSK1702934A in TRPC6 (WT or KYD)-expressing RAOSMCs. (**m**) Cumulative increases are shown as AUC. n=3 each. Data are shown as mean±SEM. *P<0.05; **P<0.01 using one-way ANOVA followed by Tukey’s post-hoc test (**b, h, k**) or the unpaired t-test (**m**, two-sided).


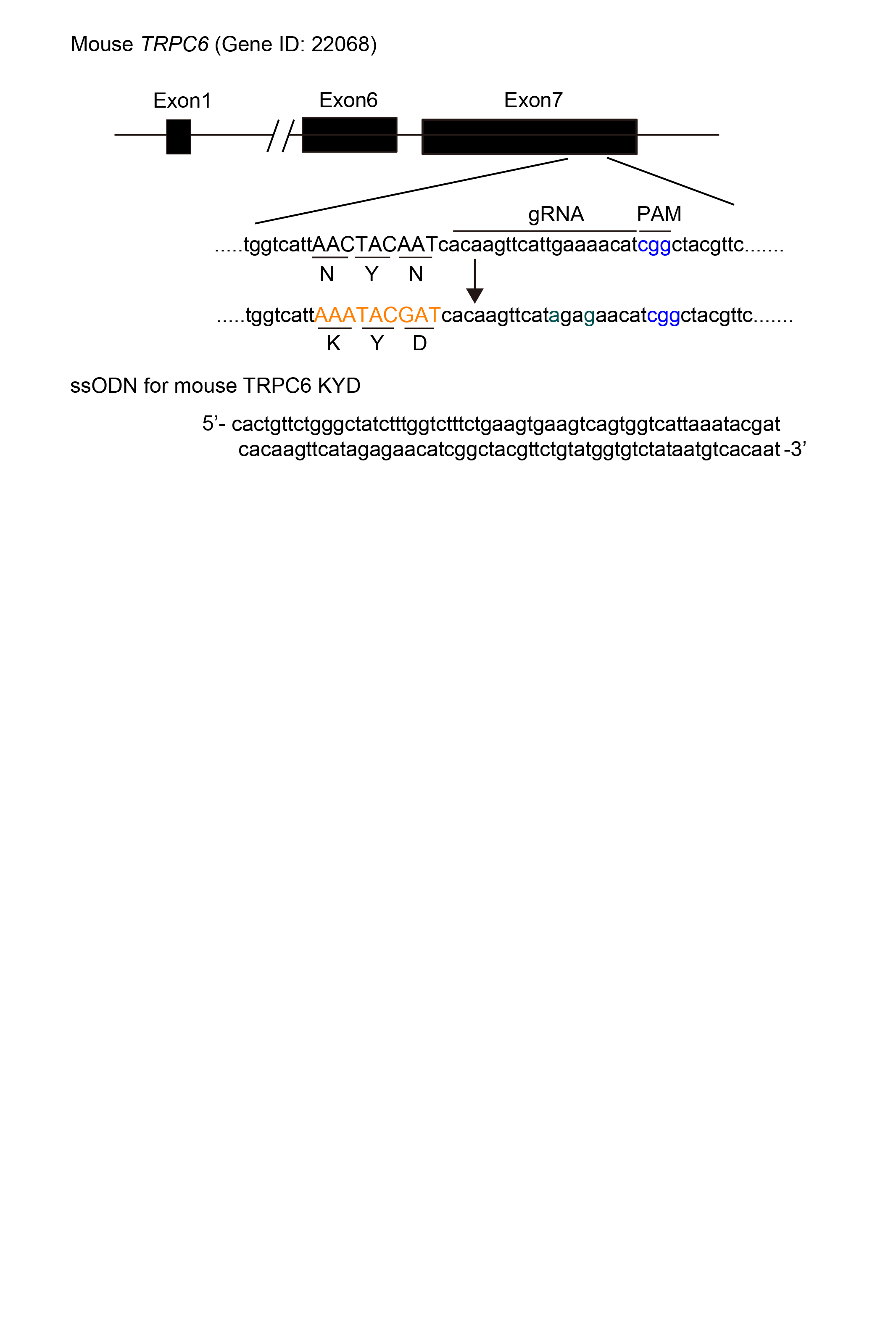


**Supplementary Figure 5. Strategy of generating TRPC6 KYD mutant mice.**

TRPC6 KYD knock-in mice (C57BL/6J) were generated by mutating the asparagine residue at position 615 to lysine (AAC to AAA) and the asparagine at position 617 to aspartic acid (AAT to GAT) using the CRISPR/Cas9 system. The single-guide RNA and template DNA were injected into oviduct. The sequence of sgRNA was 5′- cacaagttcattgaaaacat -3′ and template DNA was 5′- cactgttctgggctatctttggtctttctgaagtgaagtcagtggtcattaaatacgatcacaagttcatagagaacatcggctacgttctgtatggtgtctataatgtcacaat -3′. The single-guide RNA, template DNA and Cas9 protein were co-injected into fertilized oocytes, collected from superovulated C57BL/6J female mice mated with male mice. The next day, live zygotes were transferred into the oviduct of pseudopregnant recipient mice. To determine the genotype of pups, genomic DNA was analyzed by PCR using the following primers: F1, 5ʹ‐TTCAGGCAATTTTGATTTCTCA‐3ʹ; R1, 5ʹ‐AAGTCTTCAGCATTCCTTGACC‐3ʹ.


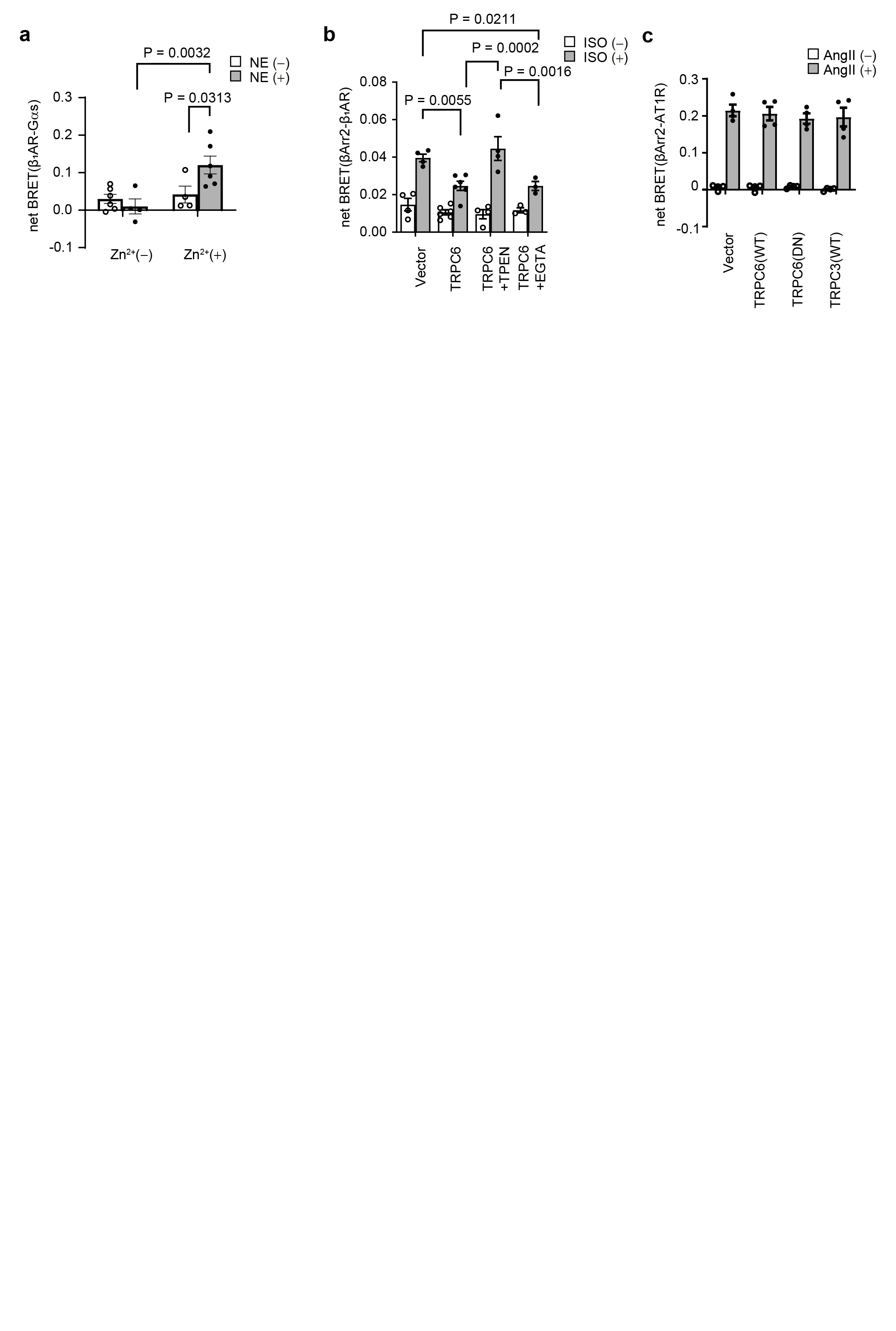


**Supplementary Figure 6. TRPC6 negatively regulates β-arrestin-dependent βAR internalization upon ISO stimulation.**

(**a**) BRET changes reflecting the association of Gα_s_ with partially activated β_1_AR in HEK293 cells expressing TRPC6, β_1_AR-Rluc and Gα_s_-YFP, with or without extracellular ZnCl_2_ (5 μM). Cells were stimulated with NE (1 μM). Zn^2+^(-) + NE (-), n=6, Zn^2+^(+) + NE (-), n=4, Zn^2+^(-) + NE (+), n=4, Zn^2+^(+) + NE (+), n=6. (**b**) BRET changes reflecting βArr2 recruitment to maximally activated β_1_AR in HEK293 cells expressing TRPC6, β_1_AR-Rluc and βArr2-YFP. Cells were pretreated with TPEN or EGTA 30 min before addition of ISO (10 μM). Vector (pCI-neo), n=3, TRPC6, n=6, TRPC6+TPEN, n=4, TRPC6+EGTA, n=3. (**c**) BRET changes reflecting βArr2 recruitment to activated AT1R in HEK293 cells expressing TRPC6 (WT or DN) or TRPC3 with AT1R-Rluc and βArr2-YFP. Cells were stimulated with Ang II (100 nM). n=4 each group. Data are shown as the mean±SEM. *P<0.05; **P<0.01 using two-way ANOVA followed by Sidak’s comparison test.


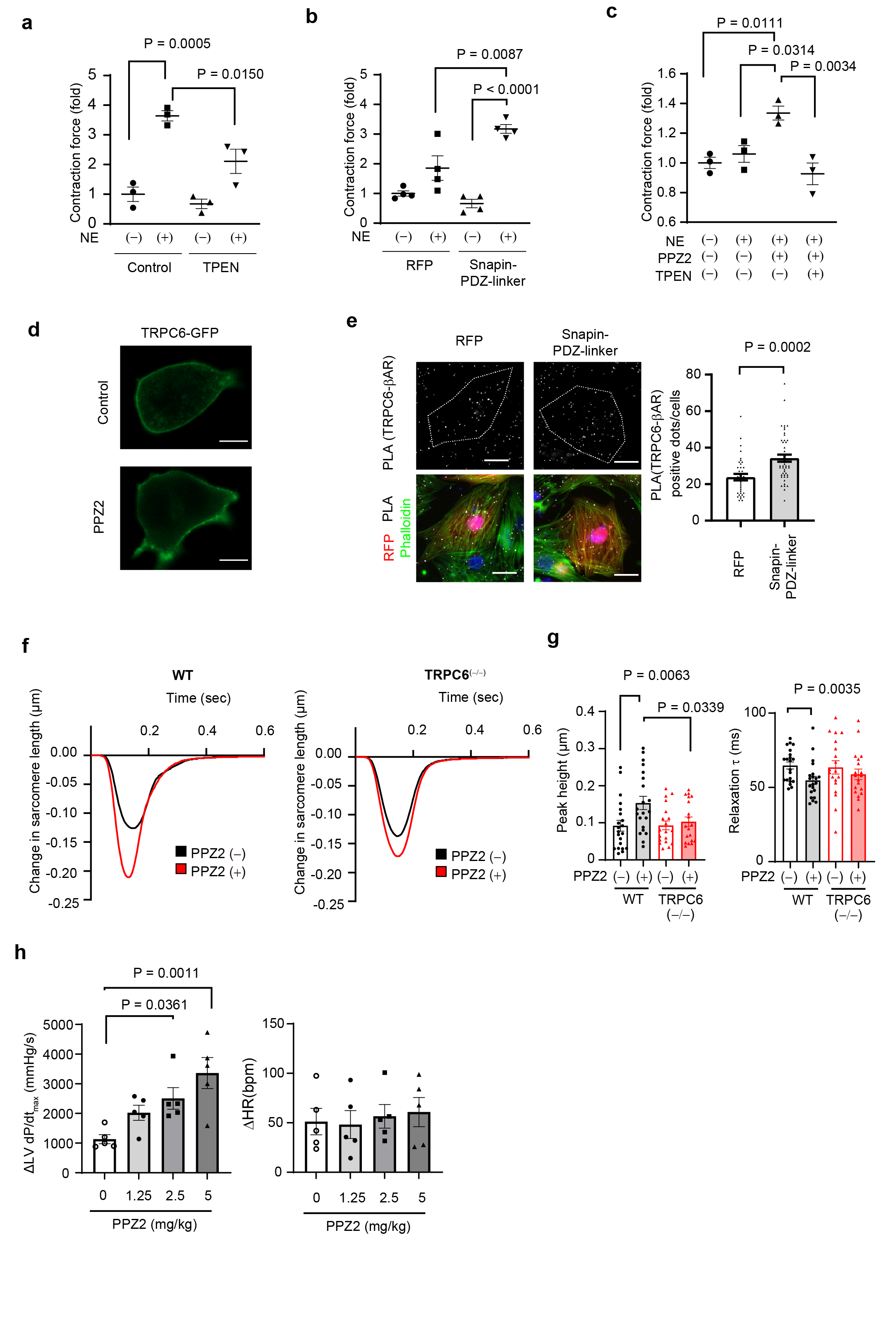


**Supplementary Figure 7. TRPC6 activator PPZ2 enhances βAR-dependent cardiomyocyte positive inotropy.**

(**a**) NE-stimulated enhancement of contractility in NRCMs pretreated with or without TPEN (25 μM) for 60 min before NE (1 μM) treatment. n=3 each group. (**b**) NE-stimulated enhancement of contractility in NRCMs expressing RFP or RFP-tagged Snapin-PDZ-linker. n=4 each. (**c**) Low-dose NE-induced contraction of NRCMs pretreated with PPZ2 (30 μM) for 30 min or TPEN (25 μM) for 60 min before stimulation with NE (0.1 μM). n=3 each. (**d**) TRPC6 localization in TRPC6-GFP-expressing HEK293 cells with or without PPZ2 (30 μM). Scale bar, 10 μm. Images are representative of three independent experiments. (**e**) Representative images of PLA of TRPC6 and β_1_AR in NRCMs expressing RFP or RFP-tagged Snapin-PDZ-linker. Scale bar, 20 μm. RFP, n=36 cells; Snapin-PDZ-linker, n=46 cells. (**f, g**) Electrical stimulation–induced contraction of adult cardiomyocytes. Representative traces of sarcomere length (**f**) and summarized results of peak heights and relaxation τ (**g**). WT, n=21 cells; TRPC6^(-/-)^, n=19 cells. (**h**) PPZ2-induced enhancement of LV ΔdP/dt max and Δheart rate following administration of hydralazine (Hyd; 0.5 mg/kg, 1.2 mL/h, *i.v.*) to mice (129/sv). n=5 each group. Data are shown as the mean±SEM. *P<0.05; **P<0.01 using the unpaired t-test (**e**, two-sided), one-way ANOVA followed by Tukey’s post-hoc test (**a, b, c**) or Dunnett's post hoc test (**h**) or two-way ANOVA followed by Sidak’s comparison test (**g**).


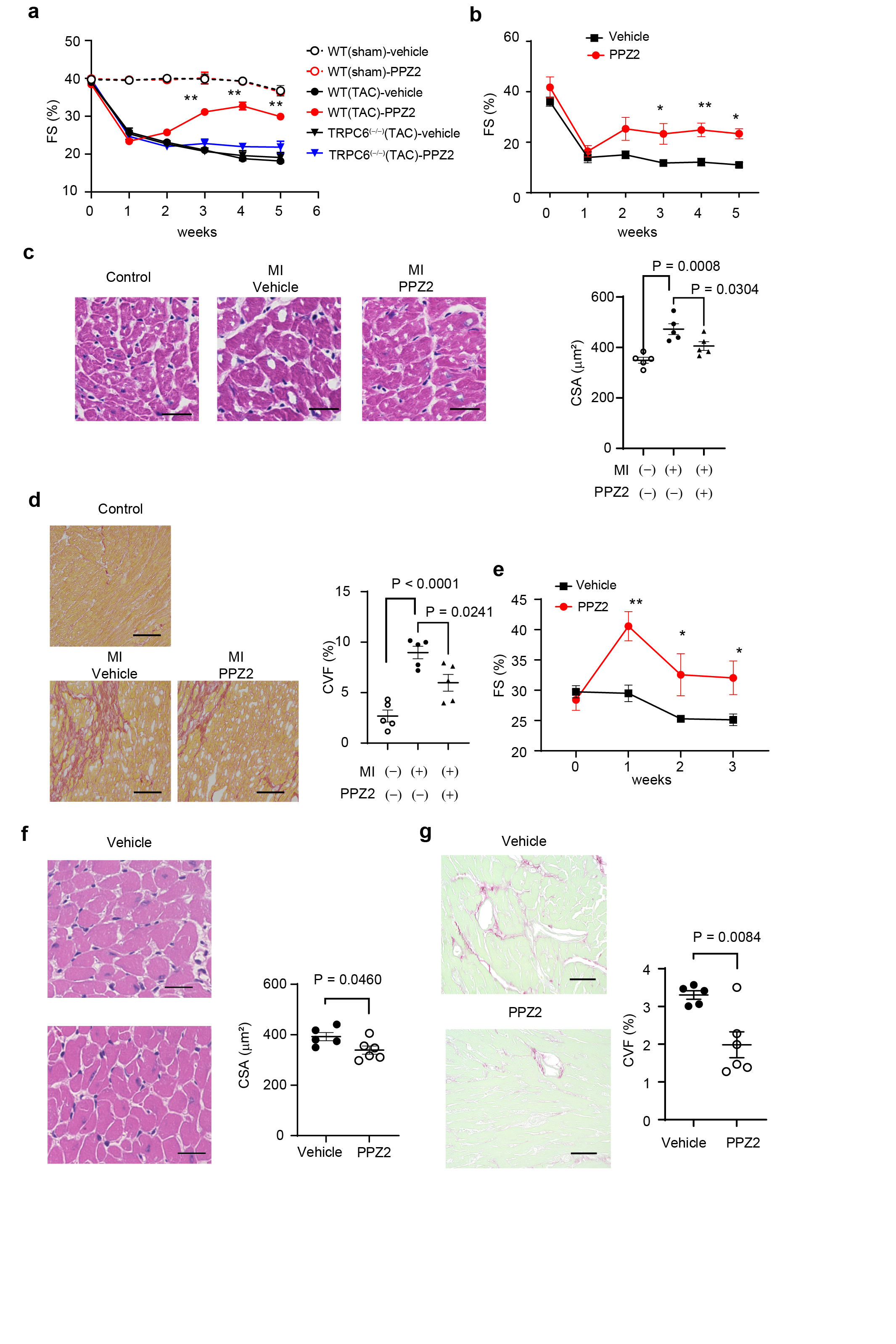


**Supplementary Figure 8. TRPC6 activator PPZ2 improves heart failure in mice.**

(**a**) Effect of PPZ2 (2.5 mg/kg/day) on the fractional shortening (FS) in TAC-operated WT and TRPC6^(-/-)^ mice (129/sv). n=5 each group; P < 0.0001 (3-5 weeks). (**b**) LV contractility of mouse (C57BL/6J) hearts after MI. n=5 each group; P = 0.0217 (3 weeks), 0.0085 (4 weeks), 0.0121 (5 weeks). (**c**) LV sections stained with hematoxylin and eosin and the quantitative results of CSA of cardiomyocytes. Scale bar, 25 μm. n=5 each. (**d**) LV sections stained with Sirius red and quantitative results of CVF. Scale bar, 100 μm. n=5 each. (**e**) LV contractility of MLP-KO mice (129/sv). n=5 each; P = 0.0020 (3 weeks), 0.0474 (4 weeks), 0.0474 (5 weeks). (**f**) LV sections stained with hematoxylin and eosin and the results of CSA. Scale bar, 25 μm. Vehicle, n=5; PPZ2, n=6. (**g**) LV sections stained with Sirius red and the results of CVF. Scale bar, 100 μm. Vehicle, n=5; PPZ2, n=6. Data are shown as mean±SEM. Significance was determined using the unpaired t-test (**f, g**), one-way ANOVA with Tukey's comparison test (**c, d**) or two-way ANOVA followed by Sidak’s comparison test (**a, b**) or Holm-Sidak’s multiple comparisons post hoc test (**e**). *P<0.05; **P<0.01.

**Supplementary Tables**

**Supplementary Table 1. Catheter left ventricular (LV)** **parameters of WT, TRPC6^(-/-)^ and TRPC3^(-/-)^ mice.**

| Genotype | Pmax (mmHg) | | | Pmin (mmHg) | | | Pmean (mmHg) | | | HR (bpm) | | |
| --- | --- | --- | --- | --- | --- | --- | --- | --- | --- | --- | --- | --- |
| WT | 107.9 | ± | 1.75 | 4.89 | ± | 1.38 | 48.2 | ± | 2.48 | 465.4 | ± | 19.0 |
| TRPC6^(-/-)^ | 123.7 | ± | 3.56 | 4.76 | ± | 0.71 | 58.0 | ± | 2.36 | 457.4 | ± | 7.1 |
| TRPC3^(-/-)^ | 115.9 | ± | 3.08 | 10.10 | ± | 2.24 | 57.5 | ± | 3.55 | 439.2 | ± | 24.9 |

| Genotype | dP/dt max (mmHg/s) | | | dP/dt min (mmHg/s) | | | Tau (ms) | | |
| --- | --- | --- | --- | --- | --- | --- | --- | --- | --- |
| WT | 6646 | ± | 326 | -7592 | ± | 600 | 9.03 | ± | 0.66 |
| TRPC6^(-/-)^ | 6690 | ± | 209 | -7147 | ± | 245 | 9.44 | ± | 0.34 |
| TRPC3^(-/-)^ | 5998 | ± | 363 | -6005 | ± | 604 | 11.85 | ± | 1.34 |

Pmax, max pressure; Pmin, minimum pressure; Pmean, mean pressure; dP/dt max, maximal rate of pressure development; dP/dt min, maximal rate of decay of pressure; tau, monoexponential time constant of relaxation. Data are shown as mean±SEM.

**Supplementary Table 2. Catheter LV parameters of mice treated with STZ.**

| Treatment | Pmax (mmHg) | | | Pmin (mmHg) | | | Pmean (mmHg) | | | HR (bpm) | | |
| --- | --- | --- | --- | --- | --- | --- | --- | --- | --- | --- | --- | --- |
| Vehicle | 117.5 | ± | 2.68 | 0.89 | ± | 2.59 | 52.2 | ± | 2.48 | 401.8 | ± | 9.86 |
| STZ | 110.0 | ± | 4.26 | 0.92 | ± | 1.72 | 47.7 | ± | 2.77 | 399.4 | ± | 12.97 |

| Treatment | dP/dt max (mmHg/s) | | | dP/dt min (mmHg/s) | | | Tau (ms) | | |
| --- | --- | --- | --- | --- | --- | --- | --- | --- | --- |
| Vehicle | 6596 | ± | 242 | -6530 | ± | 95.3 | 10.46 | ± | 1.03 |
| STZ | 6273 | ± | 356 | -6016 | ± | 345 | 10.30 | ± | 0.69 |

Pmax, max pressure; Pmin, minimum pressure; Pmean, mean pressure; dP/dt max, maximal rate of pressure development; dP/dt min, maximal rate of decay of pressure; tau, monoexponential time constant of relaxation. Data are shown as mean±SEM.

**Supplementary Table 3. Echocardiographic parameters of TAC-operated mice treated with or without PPZ2.**

|  |  |  |  |  |  |  |
| --- | --- | --- | --- | --- | --- | --- |
|  | WT (sham)-vehicle | WT (sham)-PPZ2 | WT (TAC)-vehicle | WT (TAC)-PPZ2 | TRPC6^(-/-)^ (TAC) - vehicle | TRPC6^(-/-)^ (TAC) – PPZ2 |
| Echocardiography | |  |  |  |  |  |
| n | 5 | 5 | 5 | 5 | 5 | 5 |
| EF (%) |  |  |  |  |  |  |
| week 0 | 71±1.7 | 71±2.5 | 70±1.7 | 69±2.2 | 70±2.0 | 71±0.4 |
| week 1 | 39±0.4 | 39±0.6 | 25±1.5 | 23±1.5 | 25±3.0 | 24±1.0 |
| week 2 | 40±1.0 | 39±1.8 | 23±1.3 | 25±0.9 | 22±1.4 | 22±0.8 |
| week 3 | 39±2.5 | 40±3.0 | 21±1.4 | 31±0.8 | 20±1.7 | 22±2.3 |
| week 4 | 39±1.4 | 39±1.2 | 18±1.1 | 32±2.0 | 19±2.5 | 21±1.5 |
| week 5 | 36±2.7 | 36±1.2 | 18±1.6 | 29±0.85 | 19±2.6 | 21±3.1 |
| FS (%) |  |  |  |  |  |  |
| week 0 | 39±1.2 | 39±2.0 | 39±1.5 | 38±1.8 | 38±1.6 | 39±0.3 |
| week 1 | 39±0.4 | 39±0.6 | 25±1.5 | 23±1.5 | 25±3.0 | 24±1.0 |
| week 2 | 40±1.0 | 39±1.8 | 23±1.3 | 25±0.9 | 22±1.4 | 22±0.8 |
| week 3 | 39±2.5 | 40±3.0 | 21±1.4 | 31±0.8 | 20±1.7 | 22±2.3 |
| week 4 | 39±1.4 | 39±1.2 | 18±1.1 | 32±2.0 | 19±2.5 | 21±1.5 |
| week 5 | 36.±2.7 | 36±1.2 | 18±1.6 | 29±0.8 | 19±2.6 | 21±3.1 |
| LVPWd (mm) | |  |  |  |  |  |
| week 0 | 0.68±0.04 | 0.68±0.05 | 0.64±0.03 | 0.71±0.07 | 0.69±0.07 | 0.68±0.03 |
| week 1 | 0.69±0.03 | 0.59±0.04 | 0.95±0.06 | 0.90±0.05 | 0.97±0.05 | 1.06±0.18 |
| week 2 | 0.65±0.05 | 0.63±0.03 | 0.92±0.01 | 0.89±0.120 | 1.00±0.08 | 1.05±0.15 |
| week 3 | 0.63±0.02 | 0.64±0.03 | 0.96±0.05 | 0.91±0.08 | 0.94±0.08 | 1.05±0.14 |
| week 4 | 0.64±0.05 | 0.58±0.03 | 0.96±0.08 | 0.94±0.03 | 1.09±0.08 | 1.12±0.05 |
| week 5 | 0.64±0.05 | 0.67±0.09 | 0.93±0.11 | 0.89±0.07 | 1.06±0.03 | 0.96±0.09 |
| HR (bpm) |  |  |  |  |  |  |
| week 0 | 395±59 | 417±35 | 439±23 | 405±17 | 384±25 | 380±28 |
| week 1 | 424±34 | 363±41 | 388±48 | 379±26 | 409±11 | 388±11 |
| week 2 | 416±37 | 418±25 | 415±63 | 426±52 | 405±70 | 390±28 |
| week 3 | 388±28 | 395±42 | 411±15 | 421±17 | 400±37 | 413±43 |
| week 4 | 482±38 | 394±20 | 420±25 | 427±38 | 408±10 | 435±26 |
| week 5 | 394±31 | 402±44 | 413±31 | 445±26 | 394±53 | 412±13 |
| IVSd (mm) |  |  |  |  |  |  |
| week 0 | 0.80±0.22 | 0.77±0.07 | 0.82±0.15 | 0.68±0.09 | 1.86±0.52 | 1.40±0.32 |
| week 1 | 0.86±0.15 | 0.89±0.13 | 0.89±0.11 | 1.01±0.19 | 1.40±0.32 | 1.86±0.52 |
| week 2 | 0.92±0.08 | 0.97±0.21 | 0.86±0.08 | 1.15±0.36 | 1.55±0.28 | 2.03±0.20 |
| week 3 | 1.16±0.25 | 1.27±0.19 | 1.85±0.25 | 1.55±0.51 | 1.28±0.44 | 1.29±0.24 |
| week 4 | 1.01±0.12 | 0.99±0.23 | 1.54±0.32 | 1.41±0.16 | 1.75±0.14 | 1.74±0.10 |
| week 5 | 1.43±0.34 | 1.44±0.55 | 1.35±0.26 | 1.44±0.19 | 1.67±0.29 | 1.59±0.23 |
| LVIDd (mm) |  |  |  |  |  |  |
| week 0 | 3.68±0.27 | 3.44±0.33 | 3.47±0.17 | 3.56±0.33 | 3.92±0.59 | 4.37±0.20 |
| week 1 | 3.56±0.43 | 3.59±0.39 | 3.41±0.57 | 3.63±0.19 | 4.37±0.20 | 3.92±0.59 |
| week 2 | 3.39±0.26 | 3.83±0.33 | 3.81±0.26 | 3.57±0.27 | 3.78±0.38 | 3.67±0.41 |
| week 3 | 3.76±0.31 | 3.82±0.55 | 4.02±0.23 | 3.82±0.17 | 3.81±0.49 | 4.10±0.34 |
| week 4 | 3.89±0.25 | 4.23±0.33 | 3.88±0.30 | 4.06±0.30 | 3.98±0.35 | 3.70±0.41 |
| week 5 | 4.01±0.21 | 4.01±0.35 | 4.05±0.28 | 4.32±0.34 | 4.26±0.63 | 3.86±0.37 |
| LVIDs (mm) |  |  |  |  |  |  |
| week 0 | 2.23±0.20 | 1.93±0.38 | 2.15±0.10 | 2.22±0.24 | 2.98±0.49 | 3.35±0.21 |
| week 1 | 2.15±0.27 | 2.17±0.26 | 2.76±0.39 | 2.78±0.17 | 3.35±0.21 | 2.98±0.49 |
| week 2 | 2.13±0.18 | 2.31±0.23 | 2.92±0.18 | 2.65±0.18 | 2.92±0.34 | 2.86±0.30 |
| week 3 | 2.27±0.26 | 2.30±0.42 | 3.17±0.22 | 2.81±0.14 | 3.03±0.43 | 3.17±0.34 |
| week 4 | 2.36±0.20 | 2.57±0.25 | 3.13±0.24 | 2.73±0.26 | 3.20±0.37 | 2.89±0.36 |
| week 5 | 2.54±0.22 | 2.55±0.25 | 3.31±0.25 | 3.02±0.25 | 3.41±0.54 | 3.03±0.39 |

A PPZ2-containing osmotic pump was implanted intraperitoneally 1 week after TAC (week 1) and continuous treatment was maintained for 4 weeks. HR, heart rate; IVSTd, interventricular septum diastolic thickness; LVIDd, LV internal diameter at end-diastole; LVIDs, left ventricular internal diameter at end-systole; LVPWd, left ventricular posterior wall diastolic thickness. Data are shown as mean±SEM.

**Supplementary Table 4. Body weight (BW) and heart weight (HW) of TAC-operated mice treated with or without PPZ2.**

|  | WT (sham)-vehicle | WT (sham)-PPZ2 | WT (TAC)-vehicle | WT (TAC)-PPZ2 | TRPC6^(-/-)^ (TAC) - vehicle | TRPC6^(-/-)^ (TAC) – PPZ2 |
| --- | --- | --- | --- | --- | --- | --- |
| organ weight | |  |  |  |  |  |
| n | 5 | 5 | 5 | 5 | 5 | 5 |
| BW (g) | 28.8±0.74 | 28.3±0.44 | 29.5±0.74 | 29.5±0.83 | 25.9±1.59 | 24.3±1.69 |
| HW/BW (mg/g) | 0.0038±0.000053 | 0.0041±0.00011 | 0.0050±0.00014 | 0.0050±0.00017 | 0.0056±0.00010 | 0.0059±0.00021 |

Data are shown as mean±SEM.

**Supplementary Table 5. Echocardiographic parameters of MI mice treated with or without PPZ2.**

|  | MI(+)/PPZ2(-) | MI(+)/PPZ2(+) |
| --- | --- | --- |
| Echocardiography | | |
| n | 5 | 5 |
| HR (bpm) |  |  |
| Week 0 | 510 ± 18 | 496 ± 20 |
| Week 1 | 547 ± 12 | 501 ± 13 |
| Week 2 | 531 ± 14 | 485 ± 21 |
| Week 3 | 487 ± 16 | 516 ± 19 |
| Week 4 | 526 ± 16 | 529 ± 12 |
| Week 5 | 496 ± 19 | 513 ± 19 |
| IVSd (mm) |  |  |
| Week 0 | 0.80 ± 0.02 | 0.79 ± 0.05 |
| Week 1 | 0.68 ± 0.08 | 0.70 ± 0.09 |
| Week 2 | 0.68 ± 0.06 | 0.90 ± 0.14 |
| Week 3 | 0.66 ± 0.05 | 0.65 ± 0.08 |
| Week 4 | 0.70 ± 0.11 | 0.74 ± 0.10 |
| Week 5 | 0.59 ± 0.03 | 0.84 ± 0.10 |
| LVPWd (mm) |  |  |
| Week 0 | 0.79 ± 0.03 | 0.95 ± 0.08 |
| Week 1 | 0.89 ± 0.14 | 0.82 ± 0.14 |
| Week 2 | 0.97 ± 0.12 | 0.85 ± 0.13 |
| Week 3 | 1.08 ± 0.04 | 0.82 ± 0.07 |
| Week 4 | 1.02 ± 0.09 | 0.92 ± 0.09 |
| Week 5 | 0.96 ± 0.07 | 0.75 ± 0.12 |
| LVIDd (mm) |  |  |
| Week 0 | 3.77 ± 0.12 | 3.47 ± 0.15 |
| Week 1 | 5.42 ± 0.16 | 5.07 ± 0.29 |
| Week 2 | 5.55 ± 0.20 | 4.63 ± 0.36 |
| Week 3 | 5.77 ± 0.24 | 5.08 ± 0.12 |
| Week 4 | 5.91 ± 0.16 | 4.78 ± 0.09^∗∗^ |
| Week 5 | 6.06 ± 0.14 | 5.24 ± 0.27 |
| LVIDs (mm) |  |  |
| Week 0 | 2.42 ± 0.14 | 2.04 ± 0.23 |
| Week 1 | 4.62 ± 0.17 | 4.25 ± 0.31 |
| Week 2 | 4.70 ± 0.18 | 3.52 ± 0.46 |
| Week 3 | 5.09 ± 0.24 | 3.91 ± 0.29 |
| Week 4 | 5.20 ± 0.21 | 3.59 ± 0.16^∗∗^ |
| Week 5 | 5.40 ± 0.17 | 4.03 ± 0.30^∗^ |

A PPZ2-containing osmotic pump was implanted intraperitoneally 1 week after MI (week 1) and continuous treatment was maintained for 4 weeks. HR, heart rate; IVSTd, interventricular septum diastolic thickness; LVIDd, LV internal diameter at end-diastole; LVIDs, LV internal diameter at end-systole; LVPWd, LV posterior wall diastolic thickness. Data are shown as mean±SEM. *P<0.05; **P<0.01 using the unpaired t-test.

**Supplementary Table 6. BW and HW of MI-operated** mice treated with PPZ2.

|  | MI(-)/PPZ2(-) | MI(+)/PPZ2(-) | MI(+)/PPZ2(+) |
| --- | --- | --- | --- |
| Organ weights | | |  |
| n | 5 | 5 | 5 |
| BW (g) | 22.7 ± 0.5 | 28.0 ± 0.3^##^ | 26.6 ± 0.6^##^ |
| HW (mg) | 115 ± 8 | 222 ± 13^##^ | 156 ± 6^#∗∗^ |
| HW/BW (mg/g) | 5.05 ± 0.29 | 7.92 ± 0.42^##^ | 5.87 ± 0.31^∗∗^ |
| HW/TL (g/cm) | 0.051 ± 0.003 | 0.094 ± 0.005^##^ | 0.066 ± 0.003^∗∗^ |

TL, tibia length. Data are shown as mean±SEM. Significance was determined using one-way ANOVA with Tukey’s comparison test. ^#^P<0.05, ^##^P<0.01: compared with MI(-)/PPZ2(-); ^∗∗^P<0.01: compared with MI(+)/PPZ2(-).

**Supplementary Table 7. Echocardiographic parameters of MLP-KO** mice treated with PPZ2 for 3 weeks.

|  | MLP-KO mice  Vehicle | MLP-KO mice  PPZ2 |
| --- | --- | --- |
| Echocardiography | | |
| n | 5 | 5 |
| HR (bpm) |  |  |
| Week 0 | 506 ± 3.5 | 516± 4.7 |
| Week 1 | 506 ± 8.0 | 513 ± 3.9 |
| Week 2 | 502 ± 1.7 | 523 ± 3.7 |
| Week 3 | 517± 4.9 | 511 ± 5.8 |
| IVSd (mm) |  |  |
| Week 0 | 0.68 ± 0.02 | 0.66 ± 0.02 |
| Week 1 | 0.73 ± 0.05 | 0.87 ± 0.09 |
| Week 2 | 0.71 ± 0.05 | 0.78 ± 0.06 |
| Week 3 | 0.72 ± 0.05 | 0.71 ± 0.05 |
| LVPWd (mm) |  |  |
| Week 0 | 0.77 ± 0.09 | 0.70 ± 0.04 |
| Week 1 | 0.86 ± 0.06 | 0.87 ± 0.08 |
| Week 2 | 0.78 ± 0.08 | 0.87 ± 0.09 |
| Week 3 | 0.75 ± 0.05 | 0.74 ± 0.06 |
| LVIDd (mm) |  |  |
| Week 0 | 4.19 ± 0.11 | 4.13 ± 0.18 |
| Week 1 | 4.12 ± 0.16 | 3.71 ± 0.33 |
| Week 2 | 4.59 ± 0.11 | 4.09 ± 0.08 |
| Week 3 | 4.42 ± 0.19 | 4.31 ± 0.15 |
| LVIDs (mm) |  |  |
| Week 0 | 3.00 ± 0.10 | 2.95 ± 0.15 |
| Week 1 | 2.99 ± 0.21 | 2.38 ± 0.33 |
| Week 2 | 3.51 ± 0.15 | 2.89 ± 0.22 |
| Week 3 | 3.31 ± 0.18 | 3.07 ± 0.14 |

HR, heart rate; IVSTd, interventricular septum diastolic thickness; LVIDd, LV internal diameter at end-diastole; LVIDs, LV internal diameter at end-systole; LVPWd, LV posterior wall diastolic thickness. Data are shown as mean±SEM.

**Supplementary Table 8. BW and HW of MLP-KO mice treated with PPZ2 for 3 weeks.**

|  | MLP-KO mice  Vehicle | MLP-KO mice  Vehicle |
| --- | --- | --- |
| Organ weights | | |
| n | 5 | 6 |
| BW (g) | 27.0 ± 1.5 | 28.3 ± 1.9 |
| HW (mg) | 190 ± 17 | 205 ± 17 |
| HW/BW (mg/g) | 6.99 ± 0.44 | 7.27 ± 0.39 |
| HW/TL (g/cm) | 0.094 ± 0.008 | 0.097 ± 0.008 |

TL, tibia length. Data are shown as mean±SEM.

**Supplementary Table 9. List of antibodies for immunostaining and western blot**

|  | Antigen | **Host** | **Supplier** | **Catalog no.** | **Dilution** | **Usage** |
| --- | --- | --- | --- | --- | --- | --- |
| **1st** | HCN4 | Rabbit | Alomone | APC-052 | 1:100 | IF |
|  | TRPC6 | Mouse | Santa Cruz | sc-515837 | 1:50 | PLA |
|  | β_1_AR | Rabbit | abcam | ab3442 | 1:150 | PLA |
|  | βArr2 | Mouse | Santa Cruz | sc-365445 | 1:50 | PLA |
|  | GRK5 | Rabbit | Bosterbio | PB9708 | 1:2000 | WB |
|  | GRK6 | Rabbit | Cell signaling Technology | 5878S | 1:2000 | WB |
|  | DYKDDDDK tag | Mouse | FUJIFILM | 019-22394 | 1:4000 | WB |
|  | GFP | Rabbit | Cell signaling Technology | 2037S | 1:2000 | WB |
|  | GAPDH | Rabbit | Cell signaling Technology | 2118S | 1:3000 | WB |
| **2nd** | anti-Rabbit IgG, Alexa Fluor 594 |  | Invitrogen™ | A-11037 | 1:200 | IF |
|  | anti-rabbit IgG, HRP-linked Antibody |  | Cell signaling Technology | 7074 | 1:8000 | WB |

IF, immunofluorescence; PLA, proximity ligation assay; WB, western blotting

**Supplementary Table 10. List of primer pairs for RT-PCR**

| **Primers for SYBR green** | **Sequence** |  |
| --- | --- | --- |
| Mouse β_1_AR | Forward | ATCTCTGTTTACTCAAGACCGAAAGCA |
|  | Reverse | CATACTAAGCCACACTCTCCCAACTC |
| Mouse β_2_AR | Forward | AAGTTTACATCCTCCTTAACTGGTTGG |
|  | Reverse | TAGAGTAGCCGTTCCCATAGGTTTT |
| Mouse β_3_AR | Forward | GTTGTCCTGGTGTGGATCGTGT |
|  | Reverse | ATAGGGCATGTTGGAGGCAAAG |
| Rat Adenylyl cyclase 5 (AC5) | Forward | ACCATTGTGCCCCACTCCCTGTT |
|  | Reverse | TCGTCGCCCAGGCTGTAGTTGAA |
| Rat Adenylyl cyclase 6 (AC6) | Forward | CTGCTTGTGTTCATCTCTG |
|  | Reverse | GACGCTAAGCAGTAGATCA |
| Rat Phosphodiesterase 3A (PDE3A) | Forward | TCACAGGGCCTTAACTTACAC |
|  | Reverse | GGAGCAAGAATTGGTTTGTCC |
| Rat Phosphodiesterase 4B (PDE4B) | Forward | CAGCTCATGACCCAGATAAGTGG |
|  | Reverse | GTCTGCACAATGTACCATGTTGCG |
| Rat Phosphodiesterase 4D (PDE4D) | Forward | CCTCTGACTGTTATCATGCACACC |
|  | Reverse | GATCCACATCATGTATTGCACTGGC |
| Rat TRPC6 | Forward | TCACTTGGAAGAACAGTGAAAGA |
|  | Reverse | CATCCTCAATTTCCTGGAATGAAC |
| Mouse ATF6 | Forward | TCGCCTTTTAGTCCGGTTCTT |
|  | Reverse | GGCTCCATAGGTCTGACTCC |
| Mouse CHOP | Forward | CTGGAAGCCTGGTATGAGGAT |
|  | Reverse | CAGGGTCAAGAGTAGTGAAGGT |
| Mouse PERK | Forward | AGTCCCTGCTCGAATCTTCCT |
|  | Reverse | TCCCAAGGCAGAACAGATATACC |
