# Supplementary material for: Myocardial TRPC6-mediated Zn2+ influx induces beneficial positive inotropy through β-adrenoceptors
Source: Nat Commun. 2022 Oct 26;13:6374. doi: 10.1038/s41467-022-34194-9 (PMC9606288; doi:10.1038/s41467-022-34194-9)
Supplement: Supplementary file 3 — Reporting Summary [file 41467_2022_34194_MOESM3_ESM.pdf]

nature research

Corresponding author(s): Motohiro Nishida

Last updated by author(s): Sep 15, 2022

Reporting Summary

Nature Research wishes to improve the reproducibility of the work that we publish. This form provides structure for consistency and transparency in reporting. For further information on Nature Research policies, see our Editorial Policies and the Editorial Policy Checklist.

Statistics

For all statistical analyses, confirm that the following items are present in the figure legend, table legend, main text, or Methods section.

n/a	Confirmed	
☐	☒	The exact sample size (*n*) for each experimental group/condition, given as a discrete number and unit of measurement
☐	☒	A statement on whether measurements were taken from distinct samples or whether the same sample was measured repeatedly
☐	☒	The statistical test(s) used AND whether they are one- or two-sided<br>*Only common tests should be described solely by name; describe more complex techniques in the Methods section.*
☐	☒	A description of all covariates tested
☐	☒	A description of any assumptions or corrections, such as tests of normality and adjustment for multiple comparisons
☐	☒	A full description of the statistical parameters including central tendency (e.g. means) or other basic estimates (e.g. regression coefficient) AND variation (e.g. standard deviation) or associated estimates of uncertainty (e.g. confidence intervals)
☐	☒	For null hypothesis testing, the test statistic (e.g. *F*, *t*, *r*) with confidence intervals, effect sizes, degrees of freedom and *P* value noted<br>*Give P values as exact values whenever suitable.*
☒	☐	For Bayesian analysis, information on the choice of priors and Markov chain Monte Carlo settings
☒	☐	For hierarchical and complex designs, identification of the appropriate level for tests and full reporting of outcomes
☒	☐	Estimates of effect sizes (e.g. Cohen's *d*, Pearson's *r*), indicating how they were calculated

Our web collection on statistics for biologists contains articles on many of the points above.

Software and code

Policy information about availability of computer code

Data collection	No software was used for data collection.
Data analysis	Micro array data analysis was performed with Expression console software 1.2 (Thermo Fisher). Normalization was performed with MAS5 algorithm using a set of mouse maintenance genes (Thermo Fisher).G*Power3.1.9.2 software was used to calculate the sample size for each group. Statistical analysis was performed using GraphPad Prism 9.0 (GraphPad Software, LaJolla, CA). Imaging data were analyzed using ImageJ (National Institutes of Health) software. The myocyte SL changes were recorded using IonOptix software (IonOptix Corporation)

For manuscripts utilizing custom algorithms or software that are central to the research but not yet described in published literature, software must be made available to editors and reviewers. We strongly encourage code deposition in a community repository (e.g. GitHub). See the Nature Research guidelines for submitting code & software for further information.

Data

Policy information about availability of data

All manuscripts must include a data availability statement. This statement should provide the following information, where applicable:
- Accession codes, unique identifiers, or web links for publicly available datasets
- A list of figures that have associated raw data
- A description of any restrictions on data availability

Micro array data have been deposited in NCBI's Gene Expression Omnibus (GEO) and are accessible through GEO Series accession number GSE189494.

Field-specific reporting

Please select the one below that is the best fit for your research. If you are not sure, read the appropriate sections before making your selection.

☒ Life sciences ☐ Behavioural & social sciences ☐ Ecological, evolutionary & environmental sciences

For a reference copy of the document with all sections, see nature.com/documents/nr-reporting-summary-flat.pdf

Life sciences study design

All studies must disclose on these points even when the disclosure is negative.

Sample size	G*Power3.1.9.2 software was used to calculate the sample size for each group.
Data exclusions	No data were excluded from the manuscript.
Replication	As reported in the figure legends, the findings were reliably reproduced.
Randomization	Laboratory animals were randomly assigned to experimental groups. The order of treatment administration was also randomized. All cell samples were randomly selected for experiments.
Blinding	All animal samples were studied, and analysis was carried out in a blinded manner. Other Investigations were not blinded during data acquisition. Measurements and data reported did not require subjective judgment or interpretation from the investigators.

Reporting for specific materials, systems and methods

We require information from authors about some types of materials, experimental systems and methods used in many studies. Here, indicate whether each material, system or method listed is relevant to your study. If you are not sure if a list item applies to your research, read the appropriate section before selecting a response.

Materials & experimental systems

n/a	Involved in the study
☐	☒ Antibodies
☐	☒ Eukaryotic cell lines
☒	☐ Palaeontology and archaeology
☐	☒ Animals and other organisms
☒	☐ Human research participants
☒	☐ Clinical data
☒	☐ Dual use research of concern

Methods

n/a	Involved in the study
☒	☐ ChIP-seq
☒	☐ Flow cytometry
☒	☐ MRI-based neuroimaging

Antibodies

Antibodies used

HCN4 (APC-052, Alomone), TRPC6 (sc-515837, Santa Cruz), β1AR (ab3442, abcam), βArr2 (sc-365445, Santa Cruz), GRK5 (PB9708, Bosterbio), GRK6 (5878S, Cell signaling Technology), GAPDH (2118S, Cell signaling Technology), DYKDDDDK tag (019-22394, FUJIFILM), GFP (2037S, Cell signaling Technology), anti-Rabbit IgG, Alexa Fluor 594 (A-11037, Invitrogen™), anti-rabbit IgG, HRP-linked Antibody(7074, Cell signaling Technology)

Validation

All antibodies were welch-recognised clones in the field and validated by the manufactures. These antibodies are further validated and routinely used in our lab.
Abbreviation for species reactivity: H-human, M-mouse, R-rat, Mk-monkey, B-Bovine, Pg-Pig
Abbreviation for application: WB- western blotting, IP-Immunoprecipitation, IF- Immunofluorescence, IHC- Immunohistochemistry,

HCN4 (APC-052, Alomone),
species reactivity:H, M, R
Application:ICC, IF, IHC, IP, WB
Web:https://www.alomone.com/p/anti-hcn4-2/APC-052

TRPC6 (sc-515837, Santa Cruz),
species reactivity:H, M, R
Application:WB、IHC
Web:https://www.scbt.com/ja/p/trpc6-antibody-b-10

β1AR (ab3442, abcam),
species reactivity:H, M, R

Application:WB、IF
Web:https://www.abcam.co.jp/beta-1-adrenergic-receptor-antibody-ab3442.html

βArr2 (sc-365445, Santa Cruz),
species reactivity:H, M, R
Application:WB、IHC、IF
Web:https://www.scbt.com/ja/p/beta-arrestin-2-antibody-b-4

GRK5 (PB9708,Bosterbio),
species reactivity:H, M, R
Application:IHC, WB
Web:https://www.bosterbio.com/anti-grk5-picoband-trade-antibody-pb9708-boster.html

GRK6 (5878S, Cell signaling Technology),
species reactivity:H, M, R
Application: WB
Web:https://en.cellsignal.jp/products/primary-antibodies/grk6-d1a4-rabbit-mab/5878?Ns=product.currentLot.numberOfApplications%7C1&N=354558885+4294967218+4294956287&Nrpp=1000&No=%7Boffset%7D&fromPage=plp

GAPDH (2118S, Cell signaling Technology),
species reactivity:H, M, R, Mk,B, Pg
Application:WB、IHC、IF
Web:https://www.cellsignal.jp/products/primary-antibodies/gapdh-14c10-rabbit-mab/2118

DYKDDDDK tag (019-22394, FUJIFILM),
species reactivity:All
Application: WB
Web:https://labchem-wako.fujifilm.com/jp/product/detail/W01W0101-2239.html

GFP (2037S, Cell signaling Technology)
species reactivity:All
Application: WB
Web:https://www.cellsignal.jp/products/antibody-conjugates/gfp-d5-1-rabbit-mab-hrp-conjugate/2037

Eukaryotic cell lines

Policy information about cell lines

Cell line source(s)	HEK293 (ATCC Cat# CRL-1573, RRID:CVCL_0045) was obtained from the American Type Culture Collection (ATCC).
Authentication	Cell lines were not authenticated.
Mycoplasma contamination	All cell lines were tested negatively for mycoplasma contamination.
Commonly misidentified lines (See ICLAC register)	No commonly misidentified line were used in this study.

Animals and other organisms

Policy information about studies involving animals; ARRIVE guidelines recommended for reporting animal research

Laboratory animals	TRPC6(-/-),TRPC3(-/-), MLP KO mice have been backcrossing for at least 10 generations and its genetic background is considered as similar to that of WT (129 /Sv). Because TRPC6(KYD/KYD) knockin mice were made of C57BL/6J mice, the genetic background was C57BL/6J, and to reduce the off-target effect, we backcrossed the mice at least 4 generations before use in the experiment. Male mice (8-12 weeks old) were use in this study. Sprague-Dawley rat pups (male and female) on postnatal day 1-2 were purchased from Japan SLC, Inc.
Wild animals	This study didn't involve wild animals.
Field-collected samples	This study didn't involve sample collected from field.
Ethics oversight	All protocols using mice and rats were reviewed and approved by the ethics committees at the National Institutes of Natural Sciences and Kyushu University and carried out in accordance with their guidelines.

Note that full information on the approval of the study protocol must also be provided in the manuscript.
